# A Comparative Study Between Amiodarone and Implantable Cardioverter-Defibrillator in Decreasing Mortality From Sudden Cardiac Death in High-Risk Patients: A Systematic Review and Meta-Analysis

**DOI:** 10.7759/cureus.26017

**Published:** 2022-06-16

**Authors:** Hany A Zaki, Eman Shaban, Khalid Bashir, Haris Iftikhar, Adel Zahran, Waleed Salem, Amr Elmoheen

**Affiliations:** 1 Emergency Medicine, Hamad Medical Corporation, Doha, QAT; 2 Cardiology, Al Jufairi Diagnosis and Treatment, Doha, QAT; 3 Medicine, Qatar University, Doha, QAT

**Keywords:** implantable cardioverter-defibrillator (icd), systematic review and meta-analysis, reduce mortality rate, sudden cardiac death, intravenous amiodarone

## Abstract

Sudden cardiac death (SCD) is an unexpected death that occurs within one hour of symptom onset. In the United States, sudden cardiac death is considered the leading cause of natural death, accounting for 325,000 adult patients annually. SCD is more common in adult patients (above the mid-30s) and men. The risk factors that predict SCD are categorized into clinical, sociological, genetic, and psychological. To prevent the occurrence of SCD, several treatment options, especially antiarrhythmic drugs and implantable cardioverter-defibrillator (ICD), have been used.

A literature search from 2000 to 2022 was conducted on six electronic databases: PubMed, Cochrane Library, Web of Science, Embase, ScienceDirect, and Google Scholar. The search query used Boolean expressions and keywords such as amiodarone, implantable cardioverter-defibrillator, sudden cardiac death, cardiac arrest, arrhythmic death, and all-cause mortality. The articles identified from the literature search were screened using the eligibility criteria, resulting in eight articles relevant for inclusion in the review. A meta-analysis of data from six of the included studies showed that ICD was more effective in the reduction of SCD rates, with an SCD rate of 5.97% (n = 84/1,408) observed in the ICD group compared with an SCD rate of 11.81% (n = 168/1,423) observed in the amiodarone group. The results also show that ICD was more effective in reducing all-cause mortality compared with amiodarone (odds ratio (OR): 1.36; 95% confidence interval (CI): 1.06-1.74; I^2^ = 57%; P = 0.03).

ICD treatment of high-risk patients was more effective in reducing SCD and all-cause mortality rates compared with amiodarone treatment. There is evidence that amiodarone can be used as an adjuvant treatment option, especially for patients who are not eligible for ICD treatment and those who face more adverse events. Evidence has also shown that using amiodarone with ICD treatment significantly improves survival rates compared to ICD treatment only.

## Introduction and background

Sudden cardiac death (SCD) is an unexpected death caused by loss of heart function. Generally, SCD occurs within one hour of symptom onset. In the United States alone, SCD is the leading cause of natural deaths, accounting for about 325,000 adult deaths yearly [1]. This condition is most prevalent in adult patients in their mid-30s to mid-40s and is more frequent in men (twice as often) than in women. SCD is rarely observed in children, with only 1-2 per 100,000 children affected yearly. SCD is usually the first presentation observed in about one-third of patients with coronary artery disease (CAD). A previous study has reported that approximately 75%-80% of all SCDs result from CAD with or without myocardial infarction (MI) [2] and is three times more frequent in men than in women. There has been a general misconception that SCD is a result of acute myocardial infarction (MI); however, evidence shows that acute MI accounts for about 20% of cases of SCDs. SCD is often labeled as a “major electrical accident” [2,3]. This is because most SCD cases occur as a result of electrical instability.

SCDs can be predicted using several clinical risk factors. Some of the clinical risk factors associated with an increase in SCDs include age (old age), gender (mostly male), smoking cigarettes, hypertension, diabetes mellitus, and obesity [4,5]. Despite these factors being powerful predictors of SCDs, they are not specific enough in determining the risk at an individual level because of a relatively low event rate. Sociological factors such as socioeconomic and psychological factors such as social isolation, stress, and considerable life event changes are associated with increased SCDs. Several studies have reported that the incidences of SCD are higher in patients in socioeconomically deprived areas as opposed to those in more affluent areas [6,7]. On the other hand, patients who suffered SCD in a previous study were reported to have experienced life-changing events six months before suffering SCD [8].

Several treatment options, such as antiarrhythmic drugs and implantable cardioverter-defibrillator (ICD), have been used to prevent SCD. Some of the antiarrhythmic drugs that have been used in the past to prevent SCD include sotalol, amiodarone, and dofetilide. Evidence from previous research has shown that sotalol has no significant impact on the primary prevention of SCD. For example, previous research conducted by Waldo et al. [9] among patients with recent (6-42 days) MI and symptomatic heart failure reported higher rates of death among patients in the sotalol group prompting termination of the trial. The results showed that the total mortality and arrhythmic deaths were significantly higher among patients in the sotalol group compared to placebo (5% versus 3.1% and 3.6% versus 2%, respectively). On the other hand, amiodarone has proved to be better than other antiarrhythmic drugs in the primary prevention of SCD. A previous meta-analysis of randomized clinical trials showed that amiodarone had a significant reduction in SCDs compared to other antiarrhythmic diets (RR: 0.44; 95% confidence interval (CI): 0.19-1.00; P = 0.93; I^2^ = 0%) [10]. Similarly, a meta-analysis of data from 6,500 patients reported that amiodarone significantly reduced arrhythmic deaths and had a modest beneficial effect in reducing overall mortality in high-risk patients [11].

Recently, ICD has been used in both the primary and secondary prevention of SCD. Several past clinical trials have reported that ICD is a more effective treatment for SCD among high-risk patients. For example, a previous multicenter trial conducted by Hua et al. in China reported that the overall mortality was significantly lower among patients in the ICD group compared to those in the non-ICD group (1.8% versus 9.4%, respectively) [12]. The trial also shows that no incident of SCD was observed among patients in the ICD group, while 26 deaths resulting from SCD were observed among patients in the non-ICD group. Previous trials comparing ICD to antiarrhythmic drugs have shown that ICD significantly reduces the incidence of SCD among high-risk patients. For example, a multicenter trial in which 95 and 101 patients were randomized to ICD and conventional therapy groups, respectively, reported that after a mean follow-up period of 2.3 years, the mortality rate was higher in the ICD group compared to the antiarrhythmic group (16% versus 39%, respectively).

Based on the previous research, meta-analyses comparing ICD to antiarrhythmic drugs in the reduction of SCD have been conducted; however, it is difficult to distinguish whether amiodarone is effective compared to ICD in reducing SCD in high-risk patients. Therefore, we conducted this study to compare the effectiveness of amiodarone to ICD in reducing SCD among high-risk patients. We hypothesize that ICD will significantly reduce SCD and all-cause mortality compared to amiodarone.

## Review

Methods

Literature Search and Reporting

We conducted a literature search through six electronic databases (PubMed, Cochrane Library, Web of Science, Embase, ScienceDirect, and Google Scholar) from 2000 to 2022. The search was conducted per Preferred Reporting Items for Systematic Reviews and Meta-Analyses (PRISMA) guidelines and a priori protocol from PROSPERO to identify randomized clinical trials and other primary studies relevant to our research. The search also used Boolean operators “AND” and “OR” combined with appropriate search terms. The search strategy used was as follows: (amiodarone) AND (implantable cardioverter-defibrillator OR ICD) AND (sudden cardiac death OR SCD OR sudden cardiac arrest OR arrhythmic death OR all-cause mortality). For additional studies, the reference lists of the identified studies and relevant systematic reviews and meta-analyses were scrutinized.

Eligibility Criteria

Articles identified in the mentioned electronic databases were independently screened by two reviewers using the inclusion and exclusion criteria. The inclusion criteria were as follows: studies conducted on human subjects only, articles written and published in the English language, studies that compared amiodarone and implantable cardioverter-defibrillator, studies with sudden cardiac death and all-cause mortality as the endpoints, and randomized clinical trials with a large number of participants (i.e., 10 or more patients/participants).

On the other hand, we excluded studies from this review based on the following criteria: studies conducted on animal subjects, articles written and published in languages other than English (we made this consideration because some scientific words are usually lost in translation), studies that independently compared either amiodarone or implantable cardioverter-defibrillator to other treatment options, studies that generalized antiarrhythmic drugs (i.e., studies that were unable to distinguish patients assigned to each antiarrhythmic drug and presented results in generalized form), studies that compared the cost benefits of amiodarone to implantable cardioverter-defibrillator, and case reports. Letters to the editor were also excluded from this review.

Quality Assessment

**Figure 1:**
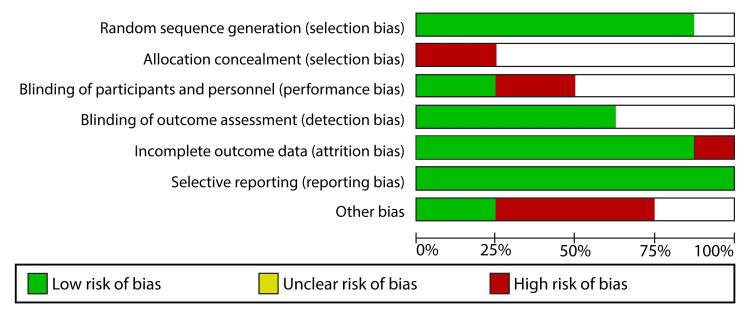
Risk of bias graph

The quality of every included study was assessed according to the criteria outlined in the Cochrane Handbook for Systematic Reviews and Meta-Analyses. The Review Manager software (RevMan 5.4.1) was instrumental in the risk of bias assessment of every study. The elements considered in assessing the study quality included selection, performance, attrition, and reporting bias. Using the mentioned elements, we classified each study into three categories: “low risk,” “high risk,” and “unclear risk.” A study was considered to have a low risk of bias if it had valid results. Therefore, studies with a low risk of bias were essential when conducting the meta-analysis of data. Studies with insufficient to invalid results were deemed to be of high risk of bias and could not be used in the meta-analysis. On the other hand, studies were deemed to be of unclear risk if the reviewers’ judgment was ambiguous due to the few details provided in the studies. Figure 1 shows the risk of bias graph.

**Figure 2:**
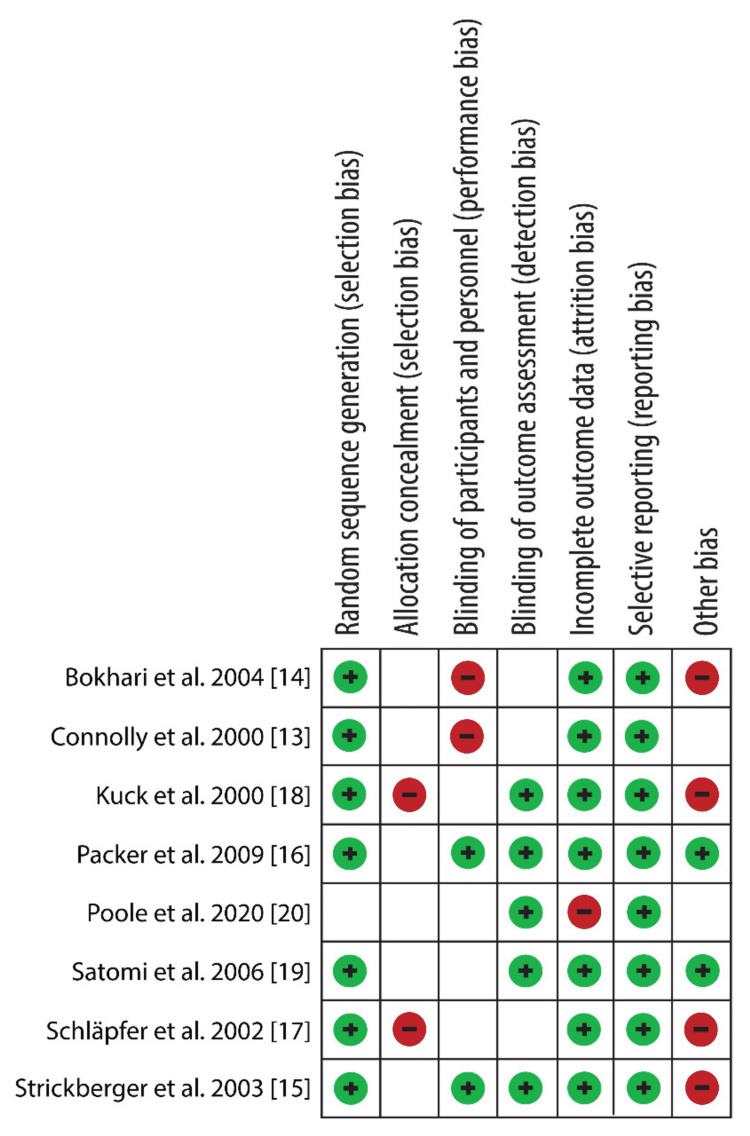
Risk of bias summary

Figure 2 shows the risk of bias summary.

Data Extraction

The data extraction process involved retrieval and compilation and was assigned to two reviewers. The data extracted from the studies included author ID, population, follow-up period, study design, intervention group, control/placebo group, and outcomes. The author ID included the authors’ names and years of publication. The primary outcome of this systematic review and meta-analysis was the number of sudden cardiac deaths, while the secondary outcome was the number of all-cause mortalities. A discussion between the two reviewers first reconciled any variation in the retrieved data. If the reviewers could not reach a consensus, a third reviewer was consulted.

Data Analysis and Synthesis

The Review Manager software (RevMan 5.4.1) was used for the meta-analysis. Since the data collected from the primary and secondary outcomes were discrete, we calculated the effect size of each outcome using the odds ratio. I^2^ statistics were used in the evaluation of study heterogeneity. Heterogeneity of greater than 50% was considered substantial. The reviewers chose to use a confidence interval of 95% because heterogeneity usually depends on the number of studies used in the meta-analysis, which was small and limited the test’s statistical power. A random-effect model was also used since it took into account both sample size and study heterogeneity. In assessing the publication bias, a funnel plot was constructed. A symmetrical funnel plot meant that there was no publication bias, while an asymmetrical funnel plot was an indication of publication bias. Forest plots were also used to show the meta-analysis of all data retrieved from the included studies.

Results

Search Results

**Figure 3:**
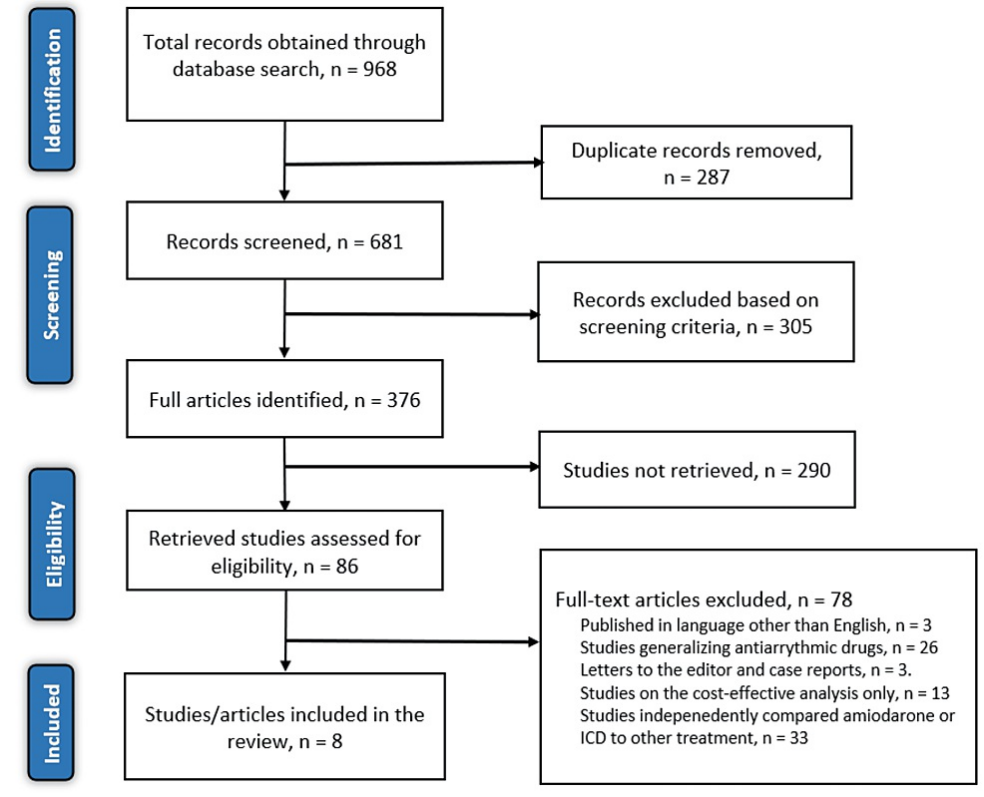
PRISMA flow diagram of the literature search results PRISMA: Preferred Reporting Items for Systematic Reviews and Meta-Analyses

After a thorough search of the six electronic databases mentioned earlier, 968 articles were identified. The two reviewers tasked with searching for relevant and original articles screened the articles for duplicates, eliminating 287 articles. The titles and abstracts of the remaining articles were then screened, excluding 305 articles that did not meet the screening criteria. Eligibility was then applied to the remaining articles, and eight articles met the criteria. The articles excluded on the basis of eligibility criteria were as follows: three articles were excluded because they were written and published in languages other than English, 33 articles were excluded because they compared either amiodarone or ICD to other treatment options, 13 articles evaluated the cost-effectiveness of the two treatment options, three articles were either systematic reviews, case reports, or letters to the editor, and 26 articles generalized antiarrhythmic drugs and did not show the outcomes for patients treated with amiodarone. Figure 3 shows the PRISMA flow diagram.

Study Characteristics

As shown in Table 1, seven randomized trials and one longitudinal study showing a comparison between amiodarone and ICD were identified and included in this systematic review and meta-analysis. The studies enrolled a total of 4,282 patients. Among all these studies, three trials employed three treatment arms, while four employed a two-treatment component. Seven out of the eight included studies were used in the meta-analysis. Six of the seven studies were used in the meta-analysis of sudden cardiac death, and all seven were used in the meta-analysis of all-cause death.

**Table 1:** Summary of study characteristics RCT: randomized controlled trial, ICD: implantable cardioverter-defibrillator

Author ID	Study design	Population	Intervention group	Control group	Mean follow-up period	Outcomes
Conolly et al., 2000 [13]	Randomized clinical trial (RCT)	The clinical trial included 659 patients.	Patients in the amiodarone group (63.8 + 9.9 mean age and 83.7% male) received ≥1,200 mg/day of amiodarone for a week, then ≥400 mg/day for more than 10 weeks, and then ≥300 mg/day.	Patients in the ICD group were implanted with an ICD with either thoracotomy or non-thoracotomy lead system.	Three years	Compared to the amiodarone group, the ICD group showed an insignificant difference in decreasing all-cause mortality rates, i.e., all-cause mortality rates in the ICD group and amiodarone group were 8.3% and 10.2% per year, respectively. The difference in arrhythmic death reduction was insignificant in the ICD and amiodarone groups. The most common adverse event observed in the amiodarone group was insomnia, with an incidence rate of 19.3%, while the most prevalent adverse event in the ICD group was ICD product discomfort with an incidence rate of 7.6%.
Bokhari et al., 2004 [14]	RCT	The trial involved 120 patients.	Sixty patients in the amiodarone group received ≥1,200 mg/day of amiodarone in the hospital and ≥400 mg/day for ≥10 weeks.	Sixty patients in the ICD group	5.6 + 2.6 years	More deaths were observed in the amiodarone group (28) than in the ICD group (16). ICD showed a significant mortality decrease per year compared to amiodarone, i.e., 5.5%/year versus 2.8%/year, respectively. Patients in the ICD group recorded fewer presumed arrhythmic deaths than those in the amiodarone group (2 versus 12, respectively). Amiodarone and ICD groups showed no significant difference in cardiac deaths (11 versus 8), vascular deaths (1 versus 1), and noncardiac deaths (4 versus 5).
Strickberger et al., 2003 [15]	RCT	The trial enrolled 103 patients (aged ≥18 years).	Fifty-two (26% female) patients in the amiodarone group received an amiodarone dosage of 800 mg/day for one week, then 400 mg/day for >1 week, and 300 mg/day for >1 year.	Fifty-one patients (33% female) in the ICD group received ICD using the conventional non-thoracotomy techniques.	2.0 + 1.3 years	The survival rates for patients randomized into the amiodarone group were 90% and 87%, while for patients in the ICD were 96% and 88% after one and three years, respectively. The arrhythmia-free survival rates for patients randomized into the amiodarone group were 82% and 73%, while for patients in the ICD group were 78% and 63% after one and three years, respectively. Sudden cardiac deaths showed an insignificant difference for patients in the amiodarone and ICD groups (2 versus 1, respectively).
Packer et al., 2009 [16]	RCT	The trial enrolled 2,521 patients (aged >18 years)	Eight hundred twenty-nine patients were randomized into the ICD group.	A total of 845 and 847 patients were randomized into the amiodarone and placebo groups, respectively.	45.5 months	The amiodarone and placebo groups recorded a high number of all-cause mortalities compared with the ICD therapy group (240 versus 244 versus 182 for amiodarone, placebo, and ICD therapy, respectively). Sudden cardiac mortality presumed to be ventricular tachyarrhythmia was observed to be high in the amiodarone (75 subjects) and placebo (95 subjects) groups compared with the ICD therapy group (37 subjects). The three groups showed a statically insignificant difference in noncardiac deaths (48 versus 54 versus 53 for ICD therapy, amiodarone, and placebo groups, respectively).
Schläpfer et al., 2002 [17]	RCT	The study enrolled 84 patients (78 men aged 21-77 years).	Forty-three patients randomized into the amiodarone group received an amiodarone dosage of 400 mg/day for three months and 200 mg/day after three months.	Forty-one patients who were nonresponsive to amiodarone were treated using ICD therapy.	63 + 30 months	Patients in the ICD group showed a significantly better global survival rate than patients in the amiodarone therapy group, i.e., ICD placement lowered the total mortality by 78%. Patients treated with ICD showed significantly lower sudden cardiac death than those treated with amiodarone (1 versus 9, respectively).
Kuck et al., 2000 [18]	RCT	The study enrolled 288 patients.	Ninety-nine patients with a mean age of 58 + 11 years (79% male) were randomized into the ICD group. Epicardial and endocardial systems were used in 55 and 44 patients, respectively.	Overall, 189 patients were randomized into the antiarrhythmic group; 92 of the 189 patients were assigned to the amiodarone group and received a dosage of 1,000 mg/day for seven days and a maintenance dosage of between 200 and 600 mg/day after that.	57 + 34 months	High crude death rates were observed in the antiarrhythmic (44.4%) group compared with the ICD group (36.4%). For crude deaths observed in the antiarrhythmic group, amiodarone accounted for 43.5%, while metoprolol accounted for 45.4%. The ICD group had significantly lower sudden deaths than the antiarrhythmic group (13% versus 33%, respectively). Amiodarone therapy accounted for 29.5% of the crude sudden deaths, while metoprolol therapy accounted for 35.1%.
Satomi et al., 2006 [19]	RCT	The study enrolled 507 patients (400 men and 107 women with a mean age of 58 + 13 years).	A total of 247 patients were randomized to the amiodarone group.	A total of 103 and 157 patients were randomized to class I antiarrhythmic and control (ICD only) groups.	38 + 27 months	The survival rate after five years was significantly higher among patients in the amiodarone group compared with patients in the class I group (86% versus 74%). After five years, the survival rates in the amiodarone and control groups showed no statistically significant difference (86% versus 77%).
Poole et al., 2020 [20]	Longitudinal study	The study analyzed data from 2,521 patients.	Overall, 845 patients were randomized to the amiodarone group.	A total of 847 and 829 patients were randomized to the placebo and ICD groups, respectively.	11 years	Of the 2,521 patients enrolled during the initial trial, 1,406 (55.8%) died during the long-term follow-up period. The 10-year mortality rate showed an insignificant difference between the three groups (52.5% versus 52.7% versus 57.2% for patients in the ICD, amiodarone, and placebo groups), respectively. Patients with ischemic heart failure showed a high mortality rate after 10 years compared with patients with nonischemic heart failure (63.7% versus 43.5%, respectively). Compared to the placebo group, ICD patients with ischemic heart failure had a significantly lower mortality rate (59.4% versus 68% for ICD and placebo, respectively).

Primary Outcome: Sudden Cardiac Death

**Figure 4:**
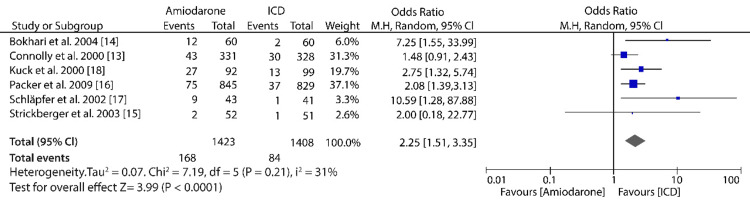
Forest plot of studies comparing amiodarone to ICD in the prevention of SCD

The pooled results of data from six randomized trials comparing amiodarone to ICD in the reduction of mortality as a result of sudden cardiac death showed that ICD treatment was more significant compared with amiodarone (OR: 2.25; 95% CI: 1.51-3.35; I^2^ = 31%; P < 0.0001) (Figure 4).

**Figure 5:**
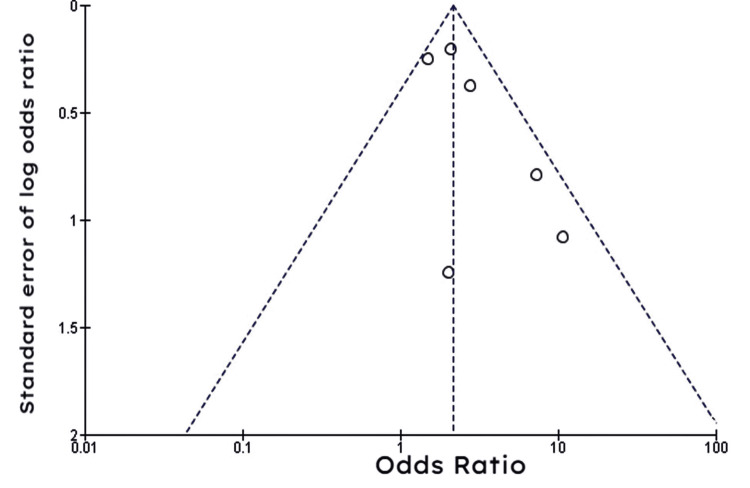
Funnel plot of studies comparing amiodarone to ICD in the prevention of SCD

Figure 5 shows a funnel plot of studies comparing amiodarone to ICD in the prevention of SCD, which was entirely symmetrical, meaning there was no evidence of publication bias.

Secondary Outcome: All-Cause Mortality

**Figure 6:**
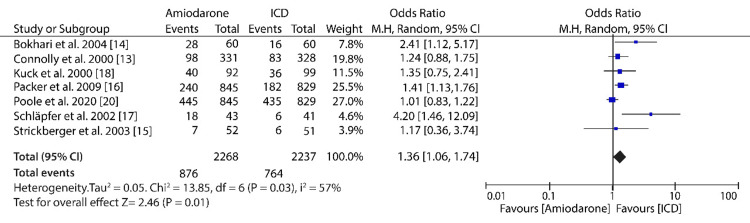
Forest plot of studies comparing amiodarone to ICD in the reduction of all-cause mortality

The pooled results of data from seven included studies showed that ICD treatment for high-risk patients was more effective in reducing the all-cause mortality compared to amiodarone treatment (OR: 1.36; 95% CI: 1.06-1.74; I^2^ = 57%; P = 0.03) (Figure 6).

**Figure 7:**
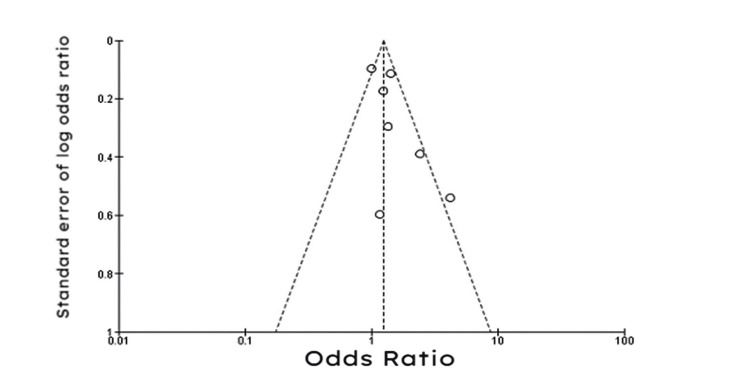
Funnel plot of studies comparing amiodarone to ICD in the reduction of all-cause mortality

The funnel plot shown in Figure 7 was largely symmetrical, meaning there was minimal evidence of publication bias.

Discussion

This review was designed to compare which treatment option between amiodarone and ICD would significantly reduce mortality from SCD among high-risk patients. The results have shown that treating high-risk patients using ICD significantly reduced the SCD mortality rate compared with the amiodarone treatment option. Similarly, ICD reduced the all-cause mortality more effectively among high-risk patients; however, the decrease observed in all-cause mortality was not as significant as in the SCD mortality rate.

Initially, we hypothesized that ICD would be more effective than amiodarone in reducing the SCD mortality rate, and the results have supported our hypothesis. Our meta-analysis results show that the SCD rate for patients treated with ICD was 5.97% (n = 84/1,408) compared with 11.81% (n = 168/1,423) SCD rate for patients treated using amiodarone. Despite showing that ICD is more effective, some of the included studies have reported that ICD and amiodarone treatments showed no significant difference in the reduction of SCD mortality rate. For example, a study conducted by Strickberger et al. [15] on 103 patients with nonischemic dilated cardiomyopathy (NIDCM) and nonsustained ventricular tachycardia (NSVT) showed that the mortality rate observed as a rate of SCD was approximately the same, i.e., out of three SCDs observed in the trial, the amiodarone group accounted for two deaths, while the ICD group accounted for one death. Similarly, a randomized controlled trial by Connolly et al. [13] reported that the arrhythmic deaths observed in the ICD and amiodarone groups were statistically insignificant. The study’s results show an insignificant reduction of arrhythmic deaths from 10.2%/year to 8.3%/year, which was observed in the ICD group. Other previous meta-analyses comparing ICD to other treatment options have also shown that ICD is an effective treatment option in reducing the SCD mortality rate. For example, a randomized multicenter trial by Hua et al. [12] reported that ICD treatment significantly reduced the SCD mortality rate compared to non-ICD treatment. The results of the trial show that no patients in the ICD group experienced SCD, while 26 patients in the non-ICD group died due to SCD. Similarly, a study conducted on 132 patients with hypertonic cardiomyopathy reported that ICD was more effective in the secondary prevention of SCD than primary prevention [21]. The study also claimed that for the primary prevention of SCD, patient selection needed to be refined.

Despite the results of our meta-analysis showing that ICD is more effective compared with amiodarone in reducing sudden cardiac death, its use may be limited by cost and technology [22]. Similarly, not every patient at high risk of sudden cardiac death is a candidate for ICD. For example, patients with New York Heart Association (NYHA) class IV heart failure and a life expectancy of less than one year are deemed noncandidates for ICD. Therefore, other pharmacological treatments such as amiodarone may be used as an alternative treatment option for these patients. Amiodarone has proved to be a more effective treatment in reducing SCD and all-cause mortality compared to other treatment options except for ICD. A previous meta-analysis of 14 randomized trials comparing amiodarone to other control treatments reported that amiodarone had a significantly lower SCD rate than placebo/control treatments [23]. The results of this meta-analysis showed that the SCD rate was 7.1% (n = 302/4,260) for patients treated with amiodarone and 9.7% (n = 413/4,262) for patients assigned to the control/placebo group. Additionally, the all-cause mortality for patients treated with ICD was statistically insignificant compared with patients in the control/placebo group (18.1% versus 19.6%). These results are similar to those recorded in a previous quantitative overview study conducted by Sim et al. [24]. The results of this study showed that the amiodarone group had a significantly lower SCD rate compared with the control groups (6.9% versus 9.6%, respectively). However, the results of this study showed that the all-cause mortality rate was significantly lower in patients treated using amiodarone compared to those assigned to the control group (16.5% versus 19.2%, respectively). Similarly, a previous meta-analysis of three randomized trials showed that amiodarone significantly lowered SCD rate compared to patients treated with other antiarrhythmic drugs (RR: 0.44; 95% CI: 0.19-1.00; I^2^ = 0; P = 0.93) [10]. The study also showed that the amiodarone group had a significantly lower all-cause mortality rate compared with antiarrhythmic drugs (RR: 0.37; 95% CI: 0.18-0.76; I^2^ = 0; P = 0.69).

On the other hand, the results of our meta-analysis have supported our hypothesis by showing that ICD lowers the all-cause mortality in high-risk patients. The results show that the all-cause mortality rate for patients treated using ICD was significantly reduced compared with patients treated using amiodarone (34.15% versus 38.62%). Other previous meta-analyses support the results of our meta-analysis. For example, a meta-analysis of two randomized trials reported that ICD had a significantly lower all-cause mortality compared to amiodarone (10.5% (n = 47/449) versus 15.1% (n = 71/471)) [25]. The rates observed in this meta-analysis are significantly lower than the results recorded in our meta-analysis. These low rates can be attributed to the fact that the meta-analysis only used two studies, and the population was much lower than the population used in our meta-analysis. Previous research studies have shown that ICD has low all-cause mortality rates compared to other treatment options. For example, a previous meta-analysis conducted on patients with nonischemic cardiomyopathy showed that ICD effectively reduced all-cause mortality. The results of this meta-analysis showed that in patients treated using ICD, the absolute mortality was reduced by 4.6% over 37.7 months (RR: 0.79; CI: 0.66-0.95; P = 0.01) [26]. Another meta-analysis conducted on patients with nonischemic cardiomyopathy reported that ICD lowered the all-cause mortality rate compared with other control groups (HR = 0.78; 95% CI: 0.66-0.92; P = 0.003). The pooled results of this analysis showed that ICD treatment in younger patients (<65 years) had a significant reduction of 47% in the incidence of total mortality [27]. Similarly, a recently updated meta-analysis on the primary prevention of dilated cardiomyopathy reported that ICD significantly reduced all-cause mortality compared to medical therapy (OR: 0.77; 95% CI: 0.64-0.93; I² = 0%; P = 0.006) [28]. Despite ICD showing efficacy in the reduction of all-cause mortality, some clinical trials have shown that the treatment has insignificant differences compared to other treatment options. A recent DANISH clinical trial reported that 120 and 131 patients in the ICD and control groups died after a median follow-up period of 67.6 months [29]. This showed a rate of 4.4 events/100 person-years and 5 events/100 person-years, which was a statistically insignificant difference.

Evidence has also shown that combining amiodarone and ICD treatment has an improved benefit in the reduction of both SCD and all-cause mortality compared to treatment using only ICD. A previous Nippon ICD Plus Pharmacological Option Necessity Study (NIPPON) included in this systematic review and meta-analysis and conducted on patients with a history of life-threatening ventricular tachyarrhythmias caused by structural heart disease reported that patients with ICD and treated using amiodarone had a significantly reduced all-cause mortality rate. The study results indicated that the total survival rates observed in the amiodarone and control groups were 86% and 77%, respectively. The arrhythmic event-free rates were also improved by treating patients with amiodarone; however, the difference was statistically insignificant (68% versus 68% at two years and 53% versus 48% at five years for the amiodarone and control groups, respectively).

On the other hand, the use of ICD and amiodarone was also associated with adverse events. Some of the adverse events observed in amiodarone treatment caused the discontinuation of the therapy and crossover to ICD treatment. According to the Cardiac Arrest Study Hamburg (CASH) [18], the most observed complication of amiodarone therapy was hyperthyroidism (3.3%). The study reported that nine patients in the amiodarone group had to discontinue treatment. The ICD group showed an overall complication rate of 23%, with perioperative death being the major complication (5.4% and 4.5% for patients with epicardial and endocardial ICD, respectively). The Canadian Implantable Defibrillator Study (CIDS) [14] reported that 19 patients in the amiodarone group had to crossover to the ICD group as a result of adverse events (n = 12) and arrhythmia recurrence (n = 7). The most common adverse event that led to the discontinuation of the amiodarone drug usage was lung-related complications (n = 5). However, the amiodarone drug was more associated with neurotoxicity (n = 26/92). The study claims that in the ICD group, no adverse event that necessitated discontinuation or crossover to the amiodarone group was observed. However, the most associated complication of ICD was the end of battery life (n = 41/50). Most of the complications observed in the ICD group were corrected by replacing the ICD. All these studies have shown that amiodarone is more associated with adverse events requiring discontinuation than ICD treatment. This argument was supported by the Amiodarone Versus Implantable Defibrillator (AMIOVIRT) study [15], which reported that 25 patients initially treated with amiodarone crossed over to ICD treatment due to adverse events compared with 11 patients in the ICD group who had to receive amiodarone treatments because of frequent defibrillator therapies (n = 1) for treatment of atrial fibrillation (n = 8) and two patients for other reasons.

Limitations of the study

The primary limitation of this review was the 57% heterogeneity observed in the studies comparing the all-cause mortality. This high heterogeneity is not uncommon and can be attributed to the fact that some studies were long-term and had large populations. Therefore, it is essential to interpret the results considerably by observing the heterogeneity. However, the heterogeneity of the included studies did not affect decision-making since the meta-analysis established that ICD was superior to amiodarone treatment in reducing all-cause mortality rates for high-risk patients. The study also did not separate the primary and secondary prevention studies; therefore, from this analysis, we are unable to identify the difference in primary and secondary prevention outcomes. Additionally, the inclusion criteria of this review only allowed studies written in English to be used, which may have led to the omission of some important and relevant studies that would have otherwise enhanced our analysis.

## Conclusions

In this systematic review and meta-analysis, treating high-risk patients using ICD more effectively reduced SCD and all-cause mortality rates than with amiodarone treatment. Despite ICD being superior, there is evidence that amiodarone can be used as an adjuvant treatment option, especially for patients who are not eligible for ICD treatment and those who face more adverse events. Evidence has also shown that using amiodarone together with ICD treatment has a significant improvement in survival rates when compared with ICD treatment only. However, the benefits in the reduction of SCD rates remain insignificant whether treated by ICD only or by ICD plus amiodarone. Amiodarone treatment was found to be more affected by adverse events; however, the clinical benefit of amiodarone in reducing SCD and all-cause mortality outweighed the adverse events, especially when compared to other non-ICD treatment options. The clinical benefits of ICD in the reduction of SCD and all-cause mortality in high-risk patients suggest that ICD can be recommended as the appropriate and effective treatment option. We would also recommend that more studies showing the clinical benefits of ICD plus amiodarone in reducing the SCD rate be conducted.
